# Uric acid and subclinical atherosclerosis: distinct associations across coronary and carotid arteries

**DOI:** 10.1007/s00392-026-02974-8

**Published:** 2026-06-29

**Authors:** Mathias Ausserwinkler, Christian Jung, Bernhard Paulweber, Johannes Bauer, Ludmilla Kedenko, Tobias Kiesslich, Barbara Fixl, Eugen Trinka, Patrick Langthaler, Bernhard Iglseder, Maria Flamm, Elmar Aigner, Bernhard Wernly

**Affiliations:** 1 Department of Internal Medicine I, University Hospital Salzburg, Paracelsus Medical University, Salzburg, Austria; 2 Department of Cardiology, Pulmonology and Vascular Medicine, Medical Faculty, Heinrich-Heine-University Duesseldorf, Duesseldorf, Germany; 3 Center of Physiology, Pathophysiology and Biophysics, Institute of Physiology and Pathophysiology, Paracelsus Medical University, Salzburg, Austria; 4 Institute of Ecomedicine, Paracelsus Medical University, Salzburg, Austria; 5 Department of Neurology, Neurointensive Care and Neurorehabilitation, Centre for Cognitive Neuroscience, Member of the European Reference Network EpiCARE, Christian Doppler University Hospital, Paracelsus Medical University, Salzburg, Austria; 6 Centre for Cognitive Neuroscience, Neuroscience Institute, Christian Doppler University Hospital, Paracelsus Medical University, Salzburg, Austria; 7 Department of Geriatric Medicine, Christian Doppler University Hospital, Paracelsus Medical University, Salzburg, Austria; 8 Institute of General Practice, Family Medicine and Preventive Medicine, Center for Public Health and Healthcare Research, Paracelsus Medical University, Salzburg, Austria

**Keywords:** Hyperuricemia, Serum Uric Acid, Gout, Coronary Artery Calcification, Carotid Plaque Burden, Subclinical Atherosclerosis

## Abstract

**Background:**

Serum uric acid has been consistently associated with cardiovascular risk, yet whether this relationship reflects independent vascular pathology or cardiometabolic risk clustering remains unresolved. We examined associations of serum uric acid and hyperuricemia with imaging-defined subclinical coronary and carotid atherosclerosis in a large population-based cohort.

**Methods:**

We analyzed data from the Paracelsus 10,000 study, a population-based cohort of adults aged 40–77 years recruited from the Austrian national population registry. Coronary artery calcium (CAC) was assessed by computed tomography in 1561 participants with available cardiac computed tomography and polygenic risk score data; carotid plaque burden was assessed by ultrasonography in 8970 participants. Associations were evaluated using ordinal logistic regression — with CAC categorized by Agatston score (0, 1–99, 100–299, ≥ 300) and carotid plaque burden categorized by total plaque area — with sequential adjustment for cardiovascular risk score (SCORE2), metabolic syndrome, polygenic cardiovascular risk, lipoprotein(a) and systemic inflammation.

**Results:**

Higher serum uric acid levels were strongly associated with greater CAC burden in unadjusted analyses (OR 1.60 per 1 mg/dL, 95% CI 1.48–1.74). This association was attenuated but remained significant after adjustment for cardiovascular risk score, metabolic syndrome, polygenic risk, lipoprotein(a), and inflammatory markers (OR 1.26, 95% CI 1.14–1.38). Hyperuricemia was independently associated with higher CAC categories after adjustment (OR 1.67, 95% CI 1.20–2.32). Carotid plaque burden showed a strong unadjusted association with serum uric acid that was substantially attenuated after multivariable adjustment, although a weak association remained statistically significant (OR 1.06, 95% CI 1.01–1.10).

**Conclusions:**

Serum uric acid and hyperuricemia are independently associated with subclinical coronary atherosclerosis beyond established cardiovascular risk factors, genetic susceptibility and systemic inflammation. The attenuation of carotid plaque associations after full adjustment suggests that extracoronary plaque burden is largely driven by cardiometabolic risk clustering rather than urate-specific pathways. These findings position uric acid as a clinically accessible marker of subclinical coronary atherosclerosis and raise the question of whether systematic urate assessment should inform cardiovascular risk stratification beyond established risk scores.

**Graphical Abstract:**

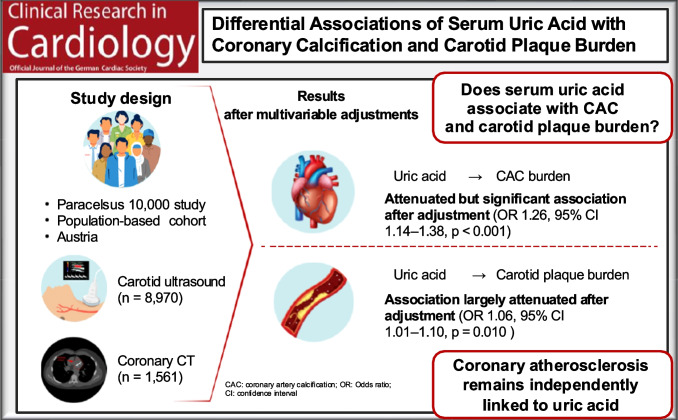

## Introduction

Cardiovascular disease remains the leading cause of death worldwide, despite progress in managing traditional risk factors such as hypertension, dyslipidemia, diabetes, and smoking [1]. Residual risk persists in many patients, prompting investigation of additional biomarkers that may contribute to atherosclerosis or reflect adverse metabolic states [2].

Serum uric acid, the end product of purine metabolism in humans, has long been associated with cardiovascular risk [3]. Unlike most mammals, humans lack functional uricase, resulting in relatively high circulating urate levels [4]. Hyperuricemia — typically defined as serum urate ≥ 6.8 mg per deciliter (the solubility threshold for monosodium urate) — is a prerequisite for gout, a chronic inflammatory condition driven by urate crystal deposition and activation of pathways such as the NLRP3 inflammasome [5, 6]. Both hyperuricemia and gout frequently cluster with cardiometabolic abnormalities, including hypertension, obesity, insulin resistance, and chronic kidney disease, complicating efforts to determine whether urate exerts independent effects on coronary heart disease [7].

Epidemiologic studies have consistently linked elevated serum uric acid to increased risks of coronary artery disease, myocardial infarction, heart failure, atrial fibrillation, and cardiovascular mortality [8]. However, the causal nature of this relationship remains uncertain. Mendelian randomization analyses support a causal role of urate in gout but provide inconsistent evidence for a direct effect on atherosclerotic cardiovascular outcomes [9]. In parallel, randomized trials of urate-lowering therapy have not consistently demonstrated reductions in major adverse cardiovascular events in patients without overt gout or severe hyperuricemia [10, 11]. Together, these findings raise the possibility that serum uric acid may function primarily as a marker of cardiometabolic risk rather than an independent causal factor.

Several biological mechanisms have been proposed to link urate to atherosclerosis, including oxidative stress related to xanthine oxidase activity, endothelial dysfunction, vascular smooth muscle proliferation and inflammatory activation [12]. Imaging studies have also identified monosodium urate deposits within vascular tissues, including coronary plaques [13]. However, these mechanistic observations do not fully resolve the discrepancy between strong epidemiologic associations and inconsistent causal evidence.

**Fig. 1:**
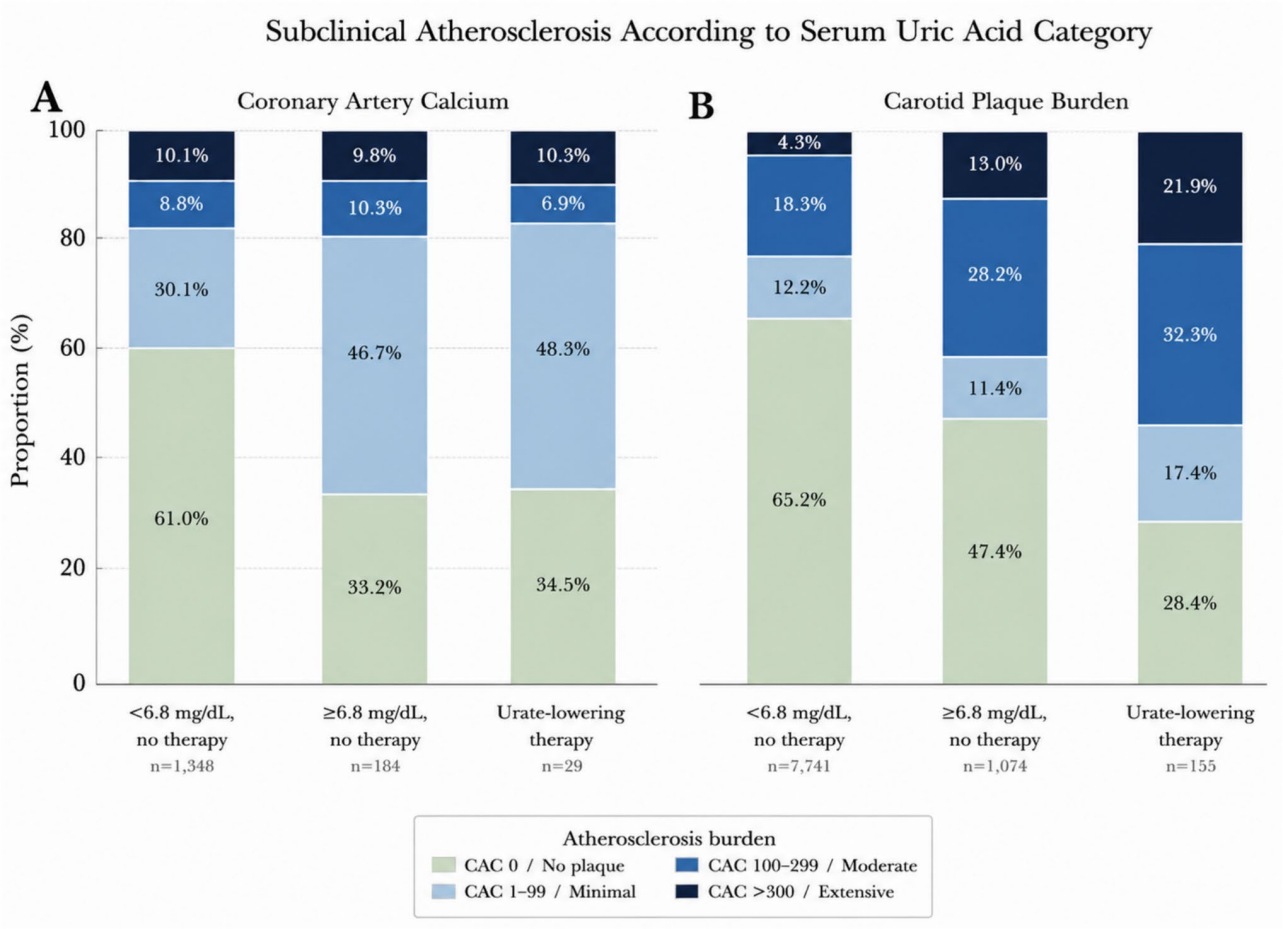
Distribution of coronary artery calcium (**A**) (CAC: 0, 1–99, 100–299 and ≥ 300) and carotid plaque burden (**B**) across serum urate groups (< 6.8 mg/dL without therapy, ≥ 6.8 mg/dL without therapy and with therapy). Percentages indicate the proportion within each group

**Fig. 2:**
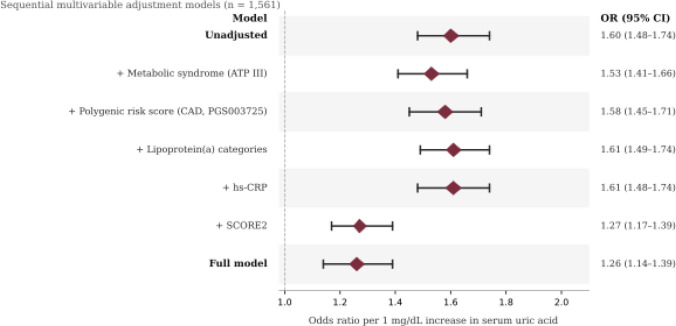
Association of serum uric acid with coronary artery calcium. Forest plot showing odds ratios (ORs) with 95% confidence intervals from ordinal logistic regression analyses assessing the association between serum uric acid (per 1 mg/dL increase) and CAC severity. Sequential models were adjusted for metabolic syndrome (ATP III), coronary artery disease polygenic risk score, lipoprotein(a) categories, inflammatory activity (hs-CRP), and SCORE2 cardiovascular risk. The fully adjusted model includes all covariates (*n* = 1561)

Most prior evidence focuses on clinical cardiovascular events rather than subclinical atherosclerosis. Coronary artery calcium (CAC), quantified by computed tomography, provides a direct, quantitative measure of cumulative coronary plaque burden and strongly predicts future events [14–16]. In contrast, less is known about whether urate-related phenotypes are differentially associated with atherosclerosis across vascular territories, particularly between coronary and extracoronary sites such as the carotid arteries. Furthermore, few studies have evaluated these associations while comprehensively accounting for multiple pathophysiological domains, including global cardiovascular risk, metabolic factors, genetic susceptibility, lipoprotein(a), and systemic inflammation (Figs. 1, 2).

We therefore investigated the associations of serum uric acid levels, hyperuricemia, and participants receiving urate-lowering therapy with imaging-defined subclinical coronary and carotid atherosclerosis in the Paracelsus 10,000 study, a population-based cohort of adults from Salzburg, Austria. Using standardized clinical, laboratory, and imaging data, we applied sequential adjustment for established cardiovascular risk (SCORE2), metabolic syndrome, polygenic risk, lipoprotein(a), and inflammation to determine whether urate-related phenotypes are independently associated with subclinical atherosclerosis or primarily reflect cardiometabolic risk clustering, and to assess potential differences between coronary and extracoronary vascular territories.

## Methods

### Study population

Potentially eligible individuals aged 40 to 77 years residing in the greater Salzburg region were contacted by invitation letters as part of the Paracelsus 10,000 study recruitment process from April 2013 to March 2020. Participants who responded and agreed to participate subsequently underwent standardized clinical, laboratory and imaging assessments according to the study protocol. The present analysis included participants with available carotid ultrasound data and, for the coronary artery calcium analysis, the subgroup with available non-contrast cardiac computed tomography, polygenic risk score data and complete covariate information. Detailed information regarding recruitment procedures, participation rates and comparisons between responders and non-responders has been published previously in the original cohort description. Participants with available coronary artery calcium imaging and complete covariate information were included in the CAC analyses. The overall cohort comprised 8970 participants with available carotid ultrasound data, whereas 1561 participants additionally underwent non-contrast cardiac computed tomography and had available polygenic risk score data. Polygenic cardiovascular risk was assessed using a validated coronary artery disease polygenic score (PGS003725), derived from genome-wide association data and standardized within the cohort.

### Clinical assessment

All participants underwent standardized examinations including medical history, anthropometric assessment, laboratory testing, and cardiovascular risk evaluation. Blood samples were obtained after an overnight fast. Measurements included lipid profiles, glucose metabolism parameters, inflammatory markers, and kidney function indices. Blood pressure was measured in the seated position after a resting period according to standardized protocols. Information on smoking status, medication use, and medical history was obtained through structured interviews. Global cardiovascular risk was assessed using the SCORE2 algorithm.

### Assessment of uric acid, hyperuricemia and urate-lowering therapy

Serum uric acid concentrations were measured from fasting blood samples using standardized enzymatic assays. Hyperuricemia was defined as a serum uric acid concentration ≥ 6.8 mg/dL, corresponding to the approximate solubility threshold of monosodium urate in serum [17]. Exploratory analyses in participants receiving urate-lowering therapy were limited by small numbers and are reported descriptively.

### Coronary artery calcium imaging

The computed tomography (CT) imaging subcohort was derived from a predefined subgroup of participants within the Paracelsus 10,000 study who underwent non-contrast cardiac computed tomography and had available polygenic risk score data. Consequently, the age distribution within this subgroup was narrower than in the overall cohort. CAC was quantified using non-contrast electrocardiographically gated cardiac computed tomography according to standardized acquisition protocols. Coronary calcification was identified as hyperattenuating lesions within the coronary arteries exceeding a threshold of 130 Hounsfield units and covering an area of at least 1 mm^2^. Agatston scores were calculated using dedicated software based on lesion density and area, in accordance with the established Agatston method. CAC burden was subsequently categorized into ordered severity groups reflecting increasing coronary atherosclerotic calcification (Agatston score 0, 1–99, 100–299 and ≥ 300), representing widely accepted risk strata for cardiovascular event prediction [14, 18].

### Carotid ultrasound assessment

Carotid atherosclerosis was assessed using high-resolution B-mode ultrasonography as part of the standardized imaging protocol of the Paracelsus 10,000 study. Bilateral ultrasound examinations of the common carotid artery, carotid bulb, internal carotid artery, and external carotid artery were performed with participants in the supine position using a dedicated vascular ultrasound system (Panasonic GM-72P00A, Panasonic Healthcare Diagnostics, USA). More than 90% of examinations were conducted by a single experienced operator following standardized acquisition procedures. Atherosclerotic plaques were defined as focal structures protruding into the arterial lumen with a diameter exceeding 1.5 mm and a plaque area greater than 2.9 mm^2^. Multiple measurements from different transducer angles were obtained to improve accuracy. Plaque morphology was classified according to the Gray–Weale classification system. Total plaque area (TPA) was calculated as the sum of all plaque surfaces detected in both carotid arteries. For the present analysis, carotid plaque burden was analyzed as an imaging marker of subclinical atherosclerosis.

### Statistical analysis

Continuous variables are presented as medians with interquartile ranges, and categorical variables as percentages. Group comparisons were performed using Wilcoxon rank-sum tests for continuous variables and chi-square tests for categorical variables.

Associations between serum uric acid measures and coronary artery calcification and carotid plaque burden were evaluated using ordinal logistic regression. Analyses were performed separately for continuous serum uric acid levels, hyperuricemia, and participants receiving urate-lowering therapy. Results are reported as odds ratios with 95% confidence intervals.

To evaluate the independence of observed associations, sequential multivariable models were prespecified across distinct pathophysiological domains. Sequential models were fitted with progressive addition of individual covariate domains: global cardiovascular risk (SCORE2), metabolic syndrome components (abdominal obesity and elevated triglycerides per ATP III criteria), polygenic cardiovascular risk (coronary artery disease polygenic score, PGS003725), lipoprotein(a) categories, and systemic inflammation (high-sensitivity C-reactive protein). A separate fully adjusted model incorporated all aforementioned covariates simultaneously, including individual metabolic syndrome components rather than the composite criterion, to avoid redundant adjustment given the partial overlap between metabolic syndrome and the SCORE2 algorithm. Alcohol consumption in grams per day was additionally included in all fully adjusted models. All fully adjusted models were additionally adjusted for statin use (binary variable).

As polygenic risk score data were available only in the imaging subcohort, sequential adjustment for carotid plaque burden did not include this covariate. This sequential approach was chosen to assess the degree to which each domain attenuates the association between uric acid and subclinical atherosclerosis. Covariates were selected a priori based on established cardiovascular risk associations and biological plausibility. As an additional genetic sensitivity analysis, three polygenic risk scores for serum uric acid (PGS000126, PGS000755, and PGS002290), derived from genome-wide association data, were evaluated in separate ordinal regression models to assess whether genetically determined variation in serum uric acid was associated with subclinical atherosclerosis.

The proportional odds assumption underlying ordinal logistic regression was formally assessed using standard diagnostic procedures and was not violated for the primary models.

All statistical analyses were conducted using StataNow/BE 19.5 for Mac (StataCorp, College Station, TX, USA).

### Ethics

The study protocol was approved by the Ethics Committee of the federal state of Salzburg (approval number 415-E/1521/3–2012, identical to previous analyses of the Paracelsus 10,000 study). All participants provided written informed consent.

## Results

A total of 8970 participants were included in the main cohort with carotid ultrasound data, and 1561 participants comprised the CT imaging subcohort with available CAC measurements and polygenic risk score data (Tables 1 and 2). Across increasing categories of serum uric acid, participants were older, more frequently male, and had a higher burden of cardiometabolic risk factors (Table 1). Body mass index, waist circumference, blood pressure and inflammatory marker increased across urate categories, as did the prevalence of metabolic syndrome, diabetes, and chronic kidney disease. Median SCORE2 cardiovascular risk rose progressively from 3.6 among normouricemic participants to 6.5 among those with hyperuricemia and 8.4 among those with urate-lowering therapy (Table 1). A similar pattern was observed in the CT imaging subcohort (Table 2). In line with the lower median LDL cholesterol levels among participants receiving urate-lowering therapy, statin use increased markedly across uric acid categories (6.7%, 13.0%, and 32.9%, respectively) and was strongly associated with higher CAC scores in the fully adjusted models.

**Table 1:** Baseline characteristics of the study population, according to serum uric acid category (main cohort)

Variable	Uric Acid < 6.8 mg/dL (*N* = 7741)	Uric Acid ≥ 6.8 mg/dL (*N* = 1074)	Urate-Lowering Therapy (*N* = 155)	*P*- value
Age — yr	54 (49–61)	57 (51–64)	63 (57–67)	< 0.001
Male sex — no. (%)	42% (3,234)	90% (963)	87% (135)	< 0.001
Body-mass index — kg/m^2^	25 (23–28)	29 (26–32)	29 (26–33)	< 0.001
Waist circumference — cm	91 (82–99)	103 (96–110)	106 (97–114)	< 0.001
Smoking status				< 0.001
Never smoker	47% (3600)	37% (397)	34% (52)	
Previous smoking	35% (2695)	47% (505)	59% (91)	
Current smoker	19% (1446)	16% (172)	8% (12)	
Alcohol consumption — g/day	7 (2–17)	14 (5–30)	15 (4–29)	< 0.001
Statin use — no. (%)	7% (520)	13% (140)	33% (51)	< 0.001
SCORE2 risk score	3.6 (1.9–6.2)	6.5 (4.2–9.7)	8.4 (5.8–11.6)	< 0.001
Metabolic syndrome — no. (%)	20% (1579)	51% (552)	67% (104)	< 0.001
Abdominal obesity — no. (%)	39% (3038)	58% (622)	62% (96)	< 0.001
Elevated triglycerides — no. (%)	21% (1646)	49% (528)	62% (96)	< 0.001
Low HDL cholesterol — no. (%)	9% (695)	19% (202)	15% (23)	< 0.001
Elevated blood pressure — no. (%)	55% (4271)	82% (885)	92% (143)	< 0.001
Elevated fasting glucose — no. (%)	23% (1756)	47% (500)	59% (91)	< 0.001
High-sensitivity CRP — mg/L	0.11 (0.06–0.22)	0.18 (0.10–0.33)	0.18 (0.09–0.35)	< 0.001
eGFR — ml/min/1.73 m^2^	90 (80–98)	83 (72–94)	79 (68–91)	< 0.001
Chronic kidney disease — no. (%)	5% (405)	12% (130)	24% (36)	< 0.001
LDL cholesterol — mg/dL	139 (116–165)	144 (118–168)	125 (93–155)	< 0.001
Lipoprotein(a) categories				0.77
Normal (0–74 nmol/L)	81% (6242)	80% (860)	83% (129)	
Borderline high (75–125 nmol/L)	6% (497)	6% (64)	6% (9)	
High (≥ 126 nmol/L)	13% (1002)	14% (150)	11% (17)	

Values are presented as medians with interquartile ranges for continuous variables and as counts with percentages for categorical variables. *P* values were calculated using the Wilcoxon rank-sum test for continuous variables and the chi-square test for categorical variables. Hyperuricemia was defined as a serum uric acid concentration of 6.8 mg per deciliter or higher in the absence of urate-lowering therapy. Metabolic syndrome was defined according to Adult Treatment Panel III criteria. Individual metabolic syndrome components are presented to reflect their use in multivariable models. The body-mass index is the weight in kilograms divided by the square of the height in meters. Estimated glomerular filtration rate was calculated using the CKD-EPI equation. Lipoprotein(a) categories were defined according to clinically relevant thresholds

**Table 2:** Baseline characteristics of the CT imaging subcohort, according to serum uric acid category

Variable	Uric Acid < 6.8 mg/dL ( *N* = 1348)	Uric Acid ≥ 6.8 mg/dL (*N* = 184)	Urate-Lowering Therapy (*N* = 29)	*P* value
Age — yr	55 (53–58)	56 (53–58)	57 (54–58)	0.069
Male sex — no. (%)	45% (606)	89% (164)	97% (28)	< 0.001
Body-mass index — kg/m^2^	25 (23–28)	29 (26–32)	30 (26–35)	< 0.001
Waist circumference — cm	91 (84–100)	103 (95–109)	106 (99–116)	< 0.001
Smoking status				0.016
Never smoker	45% (605)	36% (67)	34% (10)	
Previous smoking	36% (492)	46% (84)	59% (17)	
Current smoker	19% (251)	18% (33)	7% (2)	
Alcohol consumption — g/day	8 (2–17)	13 (4–29)	15 (6–30)	< 0.001
Statin use — no. (%)	5% (68)	12% (22)	28% (8)	< 0.001
SCORE2 risk score	3.5 (2.1–5.3)	5.3 (4.1–7.1)	6.3 (5.0–7.5)	< 0.001
Metabolic syndrome — no. (%)	19% (254)	43% (80)	69% (20)	< 0.001
Abdominal obesity — no. (%)	40% (533)	59% (109)	62% (18)	< 0.001
Elevated triglycerides — no. (%)	22% (290)	46% (85)	72% (21)	< 0.001
Low HDL cholesterol — no. (%)	9% (123)	19% (35)	14% (4)	< 0.001
Elevated blood pressure — no. (%)	49% (661)	74% (137)	79% (23)	< 0.001
Elevated fasting glucose — no. (%)	21% (280)	39% (72)	38% (11)	< 0.001
High-sensitivity CRP — mg/L	0.11 (0.06–0.20)	0.16 (0.09–0.28)	0.22 (0.12–0.35)	< 0.001
eGFR — ml/min/1.73 m^2^	90 (81–98)	84 (75–96)	87 (72–98)	< 0.001
Chronic kidney disease — no. (%)	5% (67)	9% (17)	10% (3)	0.032
LDL cholesterol — mg/dL	142 (119–168)	148 (121–173)	138 (117–168)	0.35
Lipoprotein(a) categories				0.94
Normal (0–74 nmol/L)	79% (1059)	81% (149)	76% (22)	
Borderline high (75–125 nmol/L)	7% (94)	6% (11)	7% (2)	
High (≥ 126 nmol/L)	14% (195)	13% (24)	17% (5)	
CAD polygenic risk score tertiles				0.006
Low CAD PGS (T1)	36% (481)	28% (52)	24% (7)	
Intermediate CAD PGS (T2)	34% (459)	28% (52)	38% (11)	
High CAD PGS (T3)	30% (408)	43% (80)	38% (11)	
Median Agatston CAC score (IQR)	0 (0–8)	4 (0–60)	32 (0–79)	< 0.001

Values are presented as medians with interquartile ranges for continuous variables and as counts with percentages for categorical variables. *P* values were calculated using the Wilcoxon rank-sum test for continuous variables and the chi-square test for categorical variables. The coronary artery disease polygenic risk score (PGS003725) was derived from genome-wide association data and standardized within the cohort; higher values indicate higher genetic risk. All other variables were assessed as described for Table 1. This table includes only participants with available coronary computed tomography and polygenic risk score data

In descriptive analyses, a clear gradient in coronary atherosclerosis was observed across urate categories (Table 3). The proportion of participants with any CAC (Agatston score > 0) increased from 39% among normouricemic individuals to 67% among those with hyperuricemia and 66% among those receiving urate-lowering therapy. Higher CAC categories were progressively more prevalent with increasing urate levels. A similar unadjusted pattern was observed for carotid plaque burden, with plaque present in 35% of normouricemic participants, 53% of those with hyperuricemia, and 72% of those with urate-lowering therapy (Table 3).

**Table 3:** Subclinical atherosclerosis, according to serum uric acid category

Variable	Uric Acid < 6.8 mg/dL	Uric Acid ≥ 6.8 mg/dL	Urate-Lowering Therapy	*P* value
Coronary Artery Calcification (CT Subcohort, *N* = 1561)				
Median Agatston CAC score (IQR)	0 (0–8)	4 (0–60)	32 (0–79)	< 0.001
Any CAC present (Agatston > 0) — no. (%)	39% (526)	67% (123)	66% (19)	< 0.001
Agatston score category — no. (%)				< 0.001
CAC = 0	61% (822)	33% (61)	34% (10)	
CAC 1–99	30% (406)	47% (86)	48% (14)	
CAC 100–299	4% (59)	10% (19)	7% (2)	
CAC ≥ 300	5% (61)	10% (18)	10% (3)	
Carotid Atherosclerosis (Main Cohort, N = 8970)				
Carotid plaque present — no. (%)	35% (2,694)	53% (565)	72% (111)	< 0.001
Carotid plaque burden category — no. (%)				< 0.001
No plaque (0 mm^2^)	65% (5,047)	47% (509)	28% (44)	
Minimal plaque (1–10 mm^2^)	12% (946)	11% (122)	17% (27)	
Moderate plaque (11–50 mm^2^)	18% (1,416)	28% (303)	32% (50)	
Extensive plaque (> 50 mm^2^)	4% (332)	13% (140)	22% (34)	
Carotid stenosis — no. (%)				< 0.001
No stenosis	77% (5,991)	60% (646)	43% (66)	
ECST < 50%	22% (1,717)	39% (413)	54% (84)	
ECST ≥ 50%	< 1% (25)	1% (12)	3% (5)	

Values are presented as counts with percentages. Carotid atherosclerosis was assessed in the main cohort (*N* = 8970) using high-resolution ultrasonography. Atherosclerotic plaques were defined as focal structures protruding into the arterial lumen with a diameter exceeding 1.5 mm and a plaque area greater than 2.9 mm^2^. Total plaque area was calculated as the sum of all plaque surfaces detected in both carotid arteries and categorized into predefined burden groups. Coronary artery calcification was assessed in the CT imaging subcohort (*N* = 1561) using noncontrast cardiac computed tomography. Agatston scores were calculated according to standard methods and categorized into clinically relevant strata (0, 1–99, 100–299 and ≥ 300). *P* values were calculated using the chi-square test

In unadjusted ordinal regression analyses, serum uric acid was strongly associated with both coronary artery calcium burden and carotid plaque burden (Table 4). Testing for effect modification by sex showed a significant interaction for carotid plaque burden (*P* < 0.001), with a stronger association in women than in men, while no significant interaction was found for coronary artery calcium burden (*P* = 0.767). For CAC, the odds ratio per 1 mg/dL increase in serum uric acid was 1.60 (95% CI 1.48–1.74; *P* < 0.001). For carotid plaque burden, the corresponding odds ratio was 1.44 (95% CI 1.40–1.49; *P* < 0.001).

**Table 4:** Associations of serum uric acid, hyperuricemia and urate-lowering-therapy with subclinical coronary and carotid atherosclerosis

Outcome	Exposure	Unadjusted OR (95% CI)	Model II OR (95% CI)	Model III OR (95% CI)	Model IV OR (95% CI)	Model V OR (95% CI)	Model VI OR (95% CI)	Model VII OR (95% CI)	*P* value
CAC burden	Serum uric acid (per 1 mg/dL)	1.60 (1.48–1.74)	1.53 (1.41–1.65)	1.58 (1.45–1.70)	1.61 (1.49–1.74)	1.61 (1.48–1.74)	1.27 (1.17–1.38)	1.26 (1.14–1.38)	< 0.001
CAC burden	Hyperuricemia	2.93 (2.20–3.91)	2.48 (1.84–3.33)	2.61 (1.94–3.49)	2.95 (2.21–3.94)	2.92 (2.19–3.90)	1.83 (1.35–2.47)	1.67 (1.20–2.32)	0.002
CAC burden	Therapy	2.70 (1.37–5.31)	1.95 (0.95–3.78)	2.41 (1.22–4.78)	2.68 (1.35–5.30)	2.66 (1.34–5.25)	1.40 (0.69–2.83)	1.37 (0.66–2.84)	0.397
Carotid plaque burden	Serum uric acid (per 1 mg/dL)	1.44 (1.40–1.49)	1.32 (1.28–1.36)	NA	1.44 (1.40–1.49)	1.43 (1.39–1.48)	1.07 (1.03–1.11)	1.06 (1.01–1.10)	0.010
Carotid plaque burden	Hyperuricemia	2.31 (2.04–2.61)	1.72 (1.51–1.95)	NA	2.31 (2.05–2.62)	2.25 (1.99–2.55)	1.13 (0.99–1.29)	1.06 (0.91–1.24)	0.447
Carotid plaque burden	Therapy	NA	NA	NA	NA	NA	NA	NA	NA

Values are odds ratios with 95% confidence intervals derived from ordinal logistic regression models. Serum uric acid was analyzed as a continuous variable per 1 mg per deciliter increase. Hyperuricemia was defined as serum uric acid ≥ 6.8 mg/dL without urate-lowering therapy. Model I represents the unadjusted model. Model II was adjusted for SCORE2 cardiovascular risk score. Model III was additionally adjusted for metabolic syndrome components, including abdominal obesity and elevated triglycerides according to ATP III criteria. Model IV included adjustment for the coronary artery disease polygenic risk score (PGS003725). Model V was adjusted for lipoprotein(a) categories and high-sensitivity C-reactive protein. Model VI included SCORE2 cardiovascular risk score, metabolic syndrome components, lipoprotein(a), and high-sensitivity C-reactive protein. Model VII represents the fully adjusted model and additionally included alcohol consumption (g/day) and statin use. For coronary artery calcium analyses, the coronary artery disease polygenic risk score was additionally included in the fully adjusted model. Polygenic risk score data were available only in the CT imaging subcohort; therefore, carotid plaque burden models did not include this covariate

After sequential adjustment for predefined risk domains, the association between serum uric acid and coronary artery calcium remained robust. In the fully adjusted model, which additionally accounted for statin use, higher serum uric acid levels remained independently associated with greater CAC burden (OR per 1 mg/dL 1.26, 95% CI 1.14–1.38; *P* < 0.001). Hyperuricemia was similarly and independently associated with higher CAC categories after full adjustment (OR 1.67, 95% CI 1.20–2.32; *P* = 0.002).

In contrast, the association between serum uric acid and carotid plaque burden was substantially attenuated after multivariable adjustment. In the fully adjusted model including statin use, the association with continuous serum uric acid was only marginally significant (OR per 1 mg/dL 1.06, 95% CI 1.01–1.10; *P* = 0.010), whereas hyperuricemia was no longer significantly associated with carotid plaque burden (OR 1.06, 95% CI 0.91–1.24; *P* = 0.447). Statin use itself was strongly associated with higher plaque burden in both vascular territories.

Analyses in participants receiving urate-lowering therapy were limited by small numbers and are reported descriptively only.

To address potential differences in sample composition between coronary and carotid analyses, we performed additional analyses restricted to participants with both imaging modalities available. In this restricted sample, hyperuricemia remained not significantly associated with carotid plaque burden after multivariable adjustment (odds ratio, 1.32; 95% CI, 0.94 to 1.87; *P* = 0.11). In unadjusted analyses within the same sample, serum uric acid remained associated with carotid plaque burden (odds ratio, 1.34; 95% CI, 1.24 to 1.45; *P* < 0.001), indicating that the attenuation was driven by multivariable adjustment rather than sample selection.

As an additional genetic sensitivity analysis, three polygenic risk scores for serum uric acid were examined. None of these scores was associated with coronary artery calcium burden in the CT imaging subcohort (PGS000126: odds ratio, 1.15; 95% CI, 0.85 to 1.56; *P* = 0.37; PGS000755: odds ratio, 1.05; 95% CI, 0.79 to 1.40; *P* = 0.72; PGS002290: odds ratio, 1.07; 95% CI, 0.60 to 1.91; *P* = 0.81). Similarly, no significant associations were observed with carotid plaque burden (PGS000126: odds ratio, 1.12; 95% CI, 0.83 to 1.50; *P* = 0.46; PGS000755: odds ratio, 1.01; 95% CI, 0.77 to 1.32; *P* = 0.96; PGS002290: odds ratio, 1.19; 95% CI, 0.69 to 2.06; *P* = 0.53).

## Discussion

In this population-based analysis of the Paracelsus 10,000 cohort, higher serum uric acid levels and hyperuricemia were consistently associated with a greater burden of subclinical coronary atherosclerosis, as reflected by coronary artery calcium. This association persisted after comprehensive adjustment for traditional cardiovascular risk factors, metabolic syndrome, genetic susceptibility, lipoprotein(a), and systemic inflammation. In contrast, the association with carotid plaque burden was substantially attenuated after adjustment and was no longer significant in fully adjusted models. This divergence between coronary and extracoronary atherosclerosis represents the central finding of the present study. These findings extend a large body of epidemiological evidence linking elevated uric acid levels with vascular disease. Previous cohort studies, including MESA and the Heinz Nixdorf Recall study, have reported associations between serum uric acid and coronary artery calcium burden [19, 20]. However, these studies did not simultaneously compare coronary and carotid atherosclerosis within the same cohort nor comprehensively evaluate attenuation across multiple pathophysiological domains within a unified analytical framework. The present study extends prior work through the simultaneous incorporation of polygenic cardiovascular risk, lipoprotein(a), systemic inflammation, and metabolic risk factors, allowing a more detailed assessment of whether serum uric acid reflects independent vascular pathology or broader cardiometabolic risk clustering.

Other cohort studies and meta-analyses have demonstrated associations between hyperuricemia and incident coronary artery disease, heart failure, atrial fibrillation and cardiovascular mortality [21–23]. However, most prior investigations focused on clinical outcomes rather than structural vascular disease. CAC reflects cumulative atherosclerotic burden and is among the strongest predictors of future cardiovascular events [15]. Our findings therefore suggest that the association between urate metabolism and cardiovascular risk is already detectable at the stage of subclinical coronary atherosclerosis.

The differential pattern observed between coronary calcification and carotid plaque burden provides important pathophysiological insight. Importantly, these findings should be interpreted in the context of the fundamentally different imaging modalities used to assess coronary and carotid atherosclerosis. Coronary artery calcium scoring primarily reflects calcified plaque burden and cumulative calcific remodeling within the coronary vasculature, whereas carotid ultrasonography captures total plaque burden, including non-calcified plaques. These imaging approaches may therefore represent different biological stages and phenotypes of atherosclerosis, which could partly contribute to the divergent associations observed across vascular territories.

Experimental data suggest that uric acid may promote oxidative stress, endothelial dysfunction, vascular smooth muscle proliferation, and inflammatory activation through xanthine oxidase–dependent pathways [24]. In our analyses, the persistence of the association with coronary calcification despite adjustment for high-sensitivity C-reactive protein argues against systemic inflammation as the sole mediator. Instead, these findings support the hypothesis that urate-related mechanisms may contribute to coronary vascular remodeling or reflect cumulative metabolic exposure that preferentially affects coronary calcification. Notably, the absence of an independent association with carotid plaque burden after full adjustment, including in analyses restricted to participants with both imaging modalities, suggests that extracoronary atherosclerosis is largely driven by cardiometabolic risk clustering rather than urate-specific pathways.

The genetic analyses provide an additional layer of interpretation. Despite the robust association of circulating serum uric acid with coronary artery calcium, multiple polygenic risk scores for serum uric acid were not associated with either coronary or carotid atherosclerosis. This finding is consistent with prior Mendelian randomization studies, which support a causal role of urate in gout but provide inconsistent evidence for a direct causal effect on atherosclerotic cardiovascular disease [25, 26]. Taken together, these observations argue against a simple genetically mediated causal pathway and instead suggest that serum uric acid may act as an integrated marker of cardiometabolic and vascular risk exposure rather than an isolated causal factor.

These considerations are directly relevant to the ongoing debate regarding urate-lowering therapy for cardiovascular prevention. Large, randomized trials evaluating pharmacological urate reduction have failed to demonstrate consistent reductions in major adverse cardiovascular events. In the ALL-HEART trial, allopurinol did not reduce cardiovascular outcomes among patients with ischemic heart disease [27]. Furthermore, trials comparing febuxostat with allopurinol showed cardiovascular safety but no clear cardiovascular benefit beyond gout management [28]. Current guidelines therefore recommend urate-lowering therapy primarily for symptomatic gout, while treatment of asymptomatic hyperuricemia remains controversial [29]. Emerging observational evidence, however, suggests that achieving lower serum urate levels may be associated with improved cardiovascular outcomes [30]. Our findings align with this ambiguity: serum uric acid is a strong marker of coronary atherosclerosis, yet the absence of a corresponding genetic signal raises questions about direct causality.

The present study has several strengths. It is based on a large, well-characterized population cohort with standardized imaging and comprehensive phenotyping. The simultaneous evaluation of multiple urate-related phenotypes, together with sequential adjustment across predefined pathophysiological domains, allowed a detailed assessment of attenuation patterns. The inclusion of genetic risk scores and imaging-defined endpoints further strengthens the interpretation by reducing confounding by clinical event ascertainment. Several limitations should be acknowledged. The cross-sectional design precludes causal inference, and residual confounding cannot be excluded. Serum uric acid was measured at a single time point, which may not fully capture long-term exposure. Information on urate-lowering therapy was available within the cohort. Because urate-lowering therapy is predominantly prescribed for gout in routine clinical practice, this subgroup likely consisted mainly of participants with gout. However, as therapy-based classification does not necessarily reflect clinically validated gout, analyses involving this subgroup were considered exploratory and interpreted descriptively.

In conclusion, higher serum uric acid levels and hyperuricemia are independently associated with subclinical coronary atherosclerosis, whereas associations with carotid plaque burden appear to be largely explained by cardiometabolic risk clustering. These findings position serum uric acid as a clinically accessible marker of coronary atherosclerotic burden, while underscoring the need for further longitudinal and interventional studies to clarify its causal role and potential therapeutic implications.

## Data Availability

The data that support the findings of this study are available from the Paracelsus 10,000 study. Restrictions apply to the availability of these data, which were used under license for the current study and are therefore not publicly available. Data are, however, available from the authors upon reasonable request and with permission of the Paracelsus 10,000 study board.
